# Multimodal ultrasound imaging: a method to improve the accuracy of sentinel lymph node diagnosis in breast cancer

**DOI:** 10.3389/fonc.2024.1366876

**Published:** 2024-03-25

**Authors:** Shanshan Su, Jiayi Ye, Helin Ke, Huohu Zhong, Guorong Lyu, Zhirong Xu

**Affiliations:** ^1^ Department of Ultrasound in Medicine, The Second Affiliated Hospital of Fujian Medical University, Quanzhou, China; ^2^ Department of Radiology, The Second Affiliated Hospital of Fujian Medical University, Quanzhou, China

**Keywords:** sentinel lymph node, breast cancer, multimodal ultrasound imaging, contrast enhanced ultrasound, shear wave elastography

## Abstract

**Aim:**

This study assessed the utility of multimodal ultrasound in enhancing the accuracy of breast cancer sentinel lymph node (SLN) assessment and compared it with single-modality ultrasound.

**Methods:**

Preoperative examinations, including two-dimensional ultrasound (2D US), intradermal contrast-enhanced ultrasound (CEUS), intravenous CEUS, shear-wave elastography (SWE), and surface localization, were conducted on 86 SLNs from breast cancer patients. The diagnostic performance of single and multimodal approaches for detecting metastatic SLNs was compared to postoperative pathological results.

**Results:**

Among the 86 SLNs, 29 were pathologically diagnosed as metastatic, and 57 as non-metastatic. Single-modality ultrasounds had AUC values of 0.826 (intradermal CEUS), 0.705 (intravenous CEUS), 0.678 (2D US), and 0.677 (SWE), respectively. Intradermal CEUS significantly outperformed the other methods (p<0.05), while the remaining three methods had no statistically significant differences (p>0.05). Multimodal ultrasound, combining intradermal CEUS, intravenous CEUS, 2D US, and SWE, achieved an AUC of 0.893, with 86.21% sensitivity and 84.21% specificity. The DeLong test confirmed that multimodal ultrasound was significantly better than the four single-modal ultrasound methods (p<0.05). Decision curve analysis and clinical impact curves demonstrated the superior performance of multimodal ultrasound in identifying high-risk SLN patients.

**Conclusion:**

Multimodal ultrasound improves breast cancer SLN identification and diagnostic accuracy.

## 1 Introduction

Breast cancer accounts for approximately 30% of all malignant tumors in women, with an annual increase of 0.5%. It is the most common malignant tumor among women worldwide, and there is a trend toward a younger age of onset (1, 2). Lymph node status is significantly correlated with breast cancer staging, treatment, and prognosis. The status of the sentinel lymph node (SLN) determines the subsequent treatment approach for axillary lymph nodes (3, 4). In breast cancer, cancer cells typically enter the lymphatic system and spread to the lymph nodes through lymphatic channels. The SLN is the first lymph node to receive lymphatic drainage from the primary tumor and is considered the most likely node to be invaded by cancer cells. Therefore, the SLN is important to accurately diagnose and treat patients with breast cancer (5). Currently, the SLN can be identified during surgery using blue dye, indocyanine green, radiolabeled isotopes or magnetic nano-tracers, and a qualitative diagnosis can be performed in conjunction with biopsy (6–8). However, these methods vary significantly in terms of their accuracy in detecting SLNs, and there is a risk of unnecessary adverse reactions such as tracers entering the secondary and/or tertiary lymph nodes, and lymphatic embolism and (9–11). Additionally, the use of radioactive tracers for assessing SLN has drawbacks, including mandatory licensing and surgical waste disposal (12).

Therefore, preoperative diagnosis may eliminate the need for biopsy and lymph node dissection, thereby providing valuable information for axillary treatment. Conventional preoperative imaging methods can provide diagnostic information about axillary lymph nodes; however, they are not ideal for the localization and qualitative assessment of the SLN (13, 14). Two-dimensional ultrasound (2D US) primarily distinguishes between benign and malignant lymph nodes by observing the lymph node architecture, the boundary between the cortex and medulla of lymph nodes, and the boundary with surrounding normal tissues, and by calculating aspects such as the length-to-width ratio and cortical thickness. However, information obtained solely through 2D US is relatively limited, leading to lower sensitivity and specificity (15, 16). Recent studies have shown that contrast-enhanced ultrasonography (CEUS) can locate the SLN based on its enhancement patterns and further distinguish its status. However, there is still some variation in the diagnostic efficacy of CEUS observed in different studies (17–19). The subcutaneous injection of contrast agents to locate SLN has become an established and widely used tracking method in clinical practice (3, 20). Intradermal CEUS involves the direct injection of ultrasound contrast agents into the breast tissue, followed by monitoring of their distribution and perfusion using ultrasound equipment. It is used to directly observe the position and condition of the SLN and to determine the presence of perfusion deficits or abnormalities. Its advantage lies in its ability to locate the SLN more accurately, which makes it particularly useful for superficial SLNs. Intravenous CEUS involves the injection of ultrasound contrast agents into a patient’s veins, followed by real-time monitoring using ultrasound equipment to observe the distribution of the contrast agent within the lymph nodes. Intravenous CEUS is used to determine the location, status, and blood perfusion of the SLN, aiding in the differentiation of local defects or perfusion abnormalities. Its advantage lies in providing detailed information about the local perfusion status of the SLN, which assists in diagnosis. However, intravenous CEUS is not as accurate as intradermal CEUS in locating deep SLNs and may sometimes require more advanced technical skills. Shear wave elastography (SWE) distinguishes between benign and malignant lymph nodes by quantitatively assessing tissue stiffness in real time (21). Chen et al. reported that SWE can effectively differentiate malignant lymph nodes, which exhibit significantly higher stiffness than benign lymph nodes, with a sensitivity and specificity of 92.5% and 96.7%, respectively (22). However, different devices may use different algorithms to reconstruct and analyze tissue elastography images. These algorithms can affect image quality, contrast, and resolution. Furthermore, different devices possess different ultrasound imaging qualities and spatial resolutions, which can affect the ability of elastography to resolve and accurately depict tissue elastic properties (23).

This study aimed to explore the diagnostic value of single- and multimodal ultrasonography, including 2D US, intradermal CEUS, intravenous CEUS, and SWE, in assessing the presence of SLN metastasis. The goal of this study was to provide an optimized diagnostic approach for SLN evaluation in patients with breast cancer.

## 2 Methods

Data from 90 patients who were diagnosed with breast cancer through percutaneous biopsy at the Affiliated Second Hospital of Fujian Medical University between June 2019 and December 2022 and scheduled to undergo axillary lymph node dissection surgery were continuously collected.

The inclusion criteria were: (1) patients confirmed to have breast cancer through preoperative biopsy pathology, (2) patients with breast cancer with no significant enlargement of the axillary lymph nodes upon clinical physical examination, and (3) patients without allergic reactions to contrast agents. The exclusion criteria were: (1) patients with a history of previous breast or axillary surgery, or chemotherapy and (2) patients with incomplete pathological findings.

This study was approved by the Ethics Committee of the Second Affiliated Hospital of Fujian Medical University [89-203]. All patients provided informed consent. All methods were performed in accordance with the relevant guidelines and regulations.

### 2.1 Machines and methods

#### 2.1.1 Instruments and contrast agents

A color Doppler ultrasound diagnostic apparatus (Mindray Resona 7OB, Shenzhen, China) with a superficial probe 3-11L (frequency range, 3–11 MHz) was used to perform the ultrasound examination. SonoVue (Bracco) was used as the contrast agent, which was prepared by reconstituting 5 mL of the freeze-dried powder with physiological saline to form a suspension.

#### 2.1.2 Multimodality ultrasound examination sequence and method

##### 2.1.2.1 2D US

Two-dimensional ultrasonic morphological characteristics of the SLN were recorded. Metastatic SLN was defined as the presence of the following two points: aspect ratio < 1.5, cortical thickening, lymphatic portal disappearance, and an unclear boundary between the skin and medulla.

##### 2.1.2.2 Intradermal CEUS

The patient was placed in the supine position, and the affected upper limb was externally rotated. A 0.5 mL suspension of contrast medium was injected subdermally around the areolar region at the 3, 6, 9, and 12 o’clock positions and then lightly massaged for 30 s. Lymph vessels and lymph nodes were dynamically enhanced in CEUS mode, and the CEUS enhancement mode of the SLN was recorded. The shape of the lymphatic vessels and the position of the SLN were marked on the body surface using a marker. According to the perfusion method, SLN can be divided into the following five types: Type I, uniform high enhancement; Type II, high peripheral enhancement, low lymphoid hilum enhancement, or no enhancement; Type III, diffuse uneven enhancement; Type IV, lack of local perfusion; and Type V, no contrast infusion. Types IV and V are classified as metastatic SLN.

##### 2.1.2.3 Intravenous CEUS

A section of the SLN lymphatic portal was selected after the contrast agent was cleared; if the lymphatic portal disappeared, the section with abundant blood supply was selected. SonoVue (2.4 mL) suspension was extracted and injected through the cubical vein mass, followed by 5 mL of normal saline. The perfusion of the tumor contrast agent was dynamically observed, and the dynamic images were stored for 60 s. Metastatic lymph nodes were evaluated in either the arterial phase centripetal enhancement mode (contrast agent injected from the periphery to the interior) or mixed enhancement mode (contrast agent injected from the interior and periphery simultaneously).

##### 2.1.2.4 SWE examination

**Figure 1:**
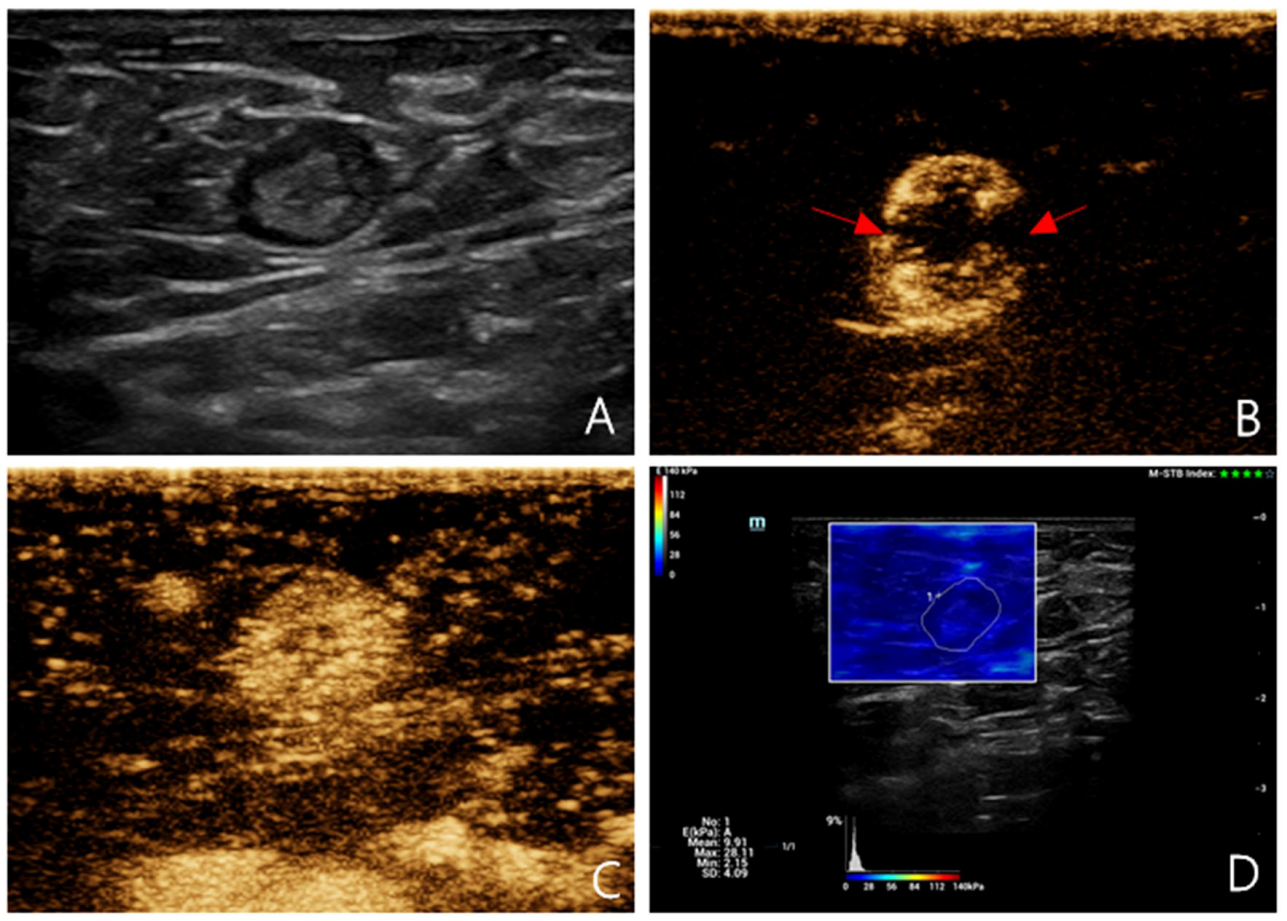
Multimodal ultrasound imaging of pathologically confirmed non-metastatic sentinel lymph node. **(A)** 2D US image; **(B)** Intradermal CEUS image (the enhancement pattern is type IV, the red arrows indicate local perfusion defect in the SLN); **(C)** Intravenous CEUS image (the enhancement pattern is mixed); **(D)** SWE image (Emean=9.91 kPa).

**Figure 2:**
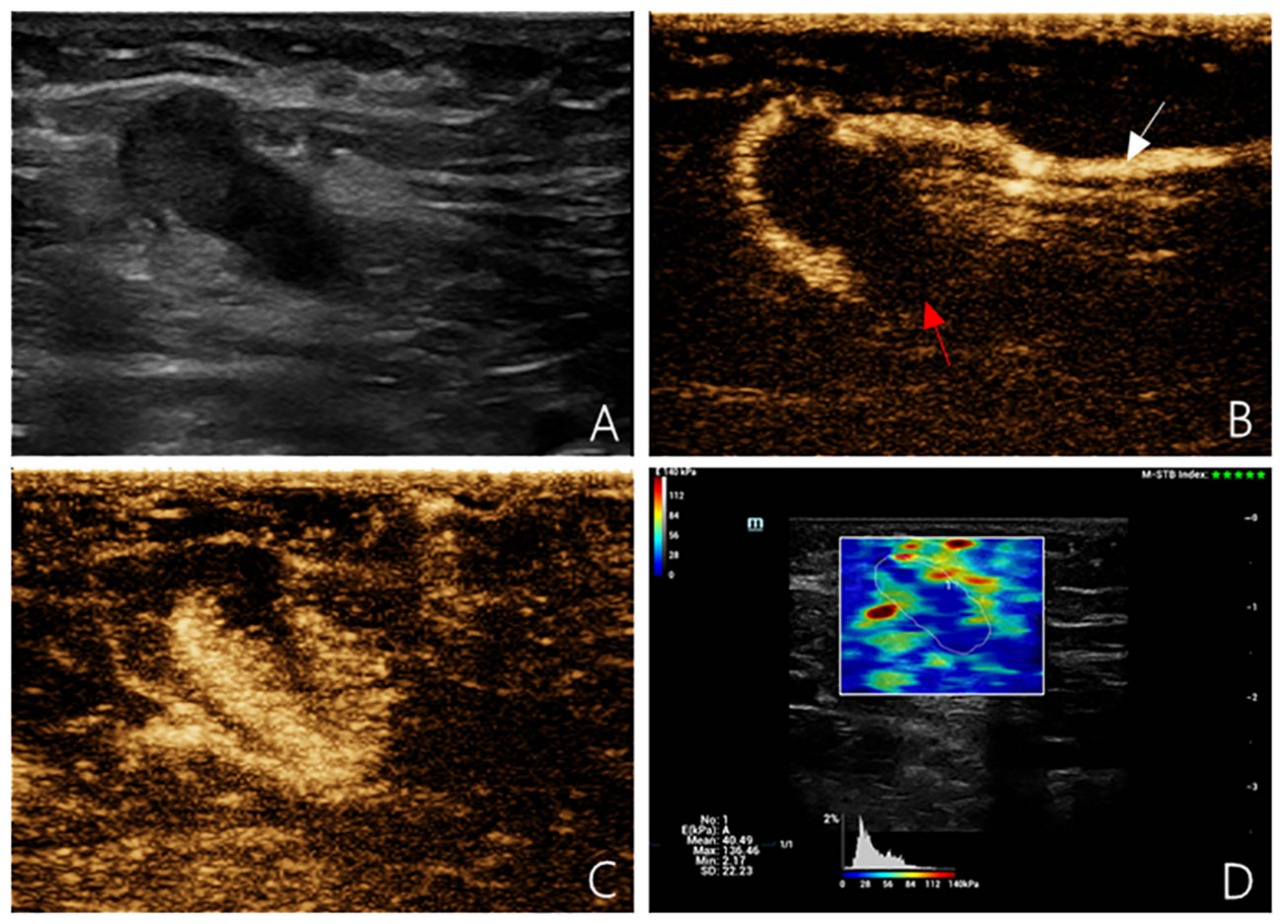
Multimodal ultrasound imaging of pathologically confirmed metastatic sentinel lymph node. **(A)** 2D US image; **(B)** Intradermal CEUS image (the enhancement pattern is type V, the white arrow indicates enhanced lymphatic duct, the red arrow indicates non-enhanced SLN); **(C)** Intravenous CEUS image (the enhancement pattern is centripetal); **(D)** SWE image (Emean=40.49 kPa).

The probe was standing, no pressure was applied, the sample frame was covered with SLN, and the image was frozen and stored after the confidence index of the sample frame exceeded 90%. Region of interest selection description method: the SLN was wrapped and its elastic modulus was measured (**Figures 1**, **2**). Based on the receiver operating characteristic (ROC) curve, the optimal SWE elasticity parameters and diagnostic threshold values were obtained to determine whether the SLN was metastatic. The SLNs with values greater than the threshold were classified as metastatic. All procedures were performed by the same experienced physician with a long history of practicing superficial ultrasound diagnostics and were recorded on video. The recorded sonograms were randomized and blindly assessed by two physicians. In cases of discrepancy, a third senior physician participated in the evaluation (**Figures 1**, **2**).

#### 2.1.3 Evaluation criteria for metastatic SLNs

Each patient underwent standardized steps during the surgical procedure to ensure accurate assessment of the SLN status. (1) Injection of Contrast Agent: During surgery, 4 mL of 1% methylene blue solution was subcutaneously injected into the tumor bed of the affected breast. This process is intended to guide the ultrasound contrast agents into the lymphatic system. (2) Breast Massage: Following the injection of the contrast agent, the breast was massaged to facilitate even distribution of the contrast agent within the breast tissue and aid its entry into the lymphatic vessels. (3) Lymphatic Duct Blue Staining: Blue dye was used to mark the lymphatic ducts. This step helps visualize the pathways of the lymphatic ducts and guides the localization of the SLN. (4) Surface Localization of Lymphatic Ducts: Before surgery, the drainage pathways of the lymphatic ducts were identified and marked using surface localization techniques. This provided crucial information for precise SLN localization. (5) Pathological examination of SLN: Localized SLN were subjected to pathological examination to check for the presence of cancer cells. This assessment helps determine the status of the SLN and whether it was affected by cancer cells.

By implementing these standardized steps, the SLNs of each patient were accurately and consistently assessed. This process ensured the reliability and comparability of the research results regarding SLN status evaluation.

### 2.2 Statistical analysis

Statistical analyses were conducted using R language software version 4.2.2 (R Foundation for Statistical Computing, Vienna, Austria). Using the SLN pathological results as the reference, ROC curves were generated to evaluate the diagnostic performance of single-modal ultrasound (2D US, intradermal CEUS, intravenous CEUS, and SWE) and multimodal ultrasound for assessing SLN characteristics. A comparison of the areas under the curve (AUC) among the different methods was performed using the DeLong method. Differences were considered statistically significant at P<0.05.

## 3 Results

### 3.1 Basic characteristics of the enrolled patients and ultrasound examinations results

**Figure 3:**
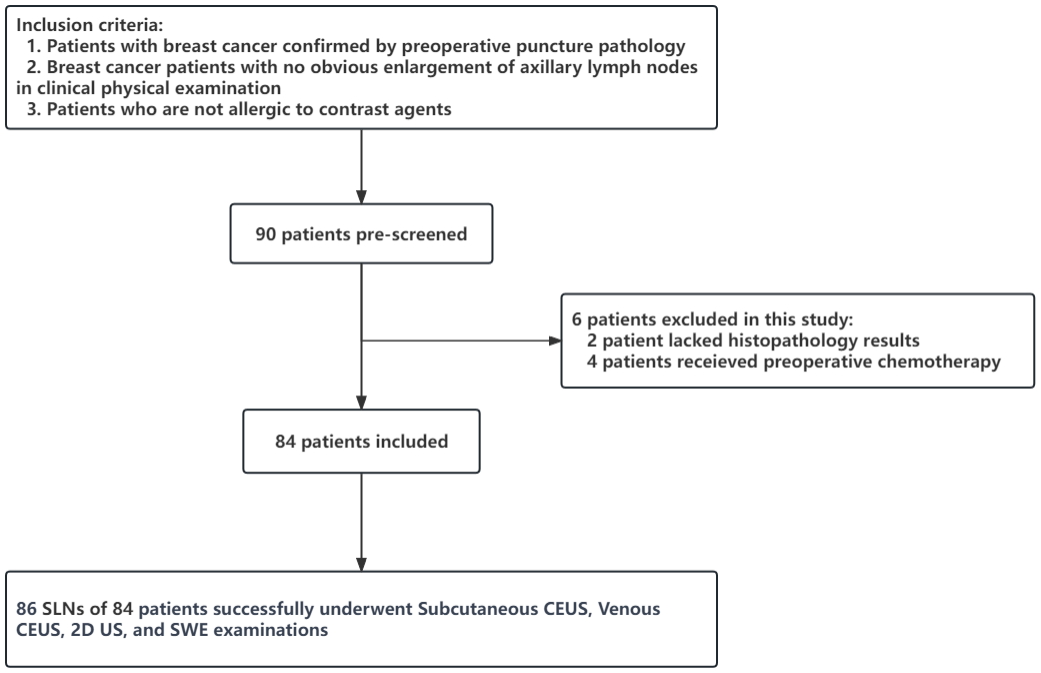
Flowchart of participant of inclusion/exclusion.

This study included a total of 90 patients with breast cancer. Among them, two were excluded because of incomplete pathological findings, and four were excluded because their preoperative treatment plans changed to neoadjuvant chemotherapy. Ultimately, 84 patients were included in the study (**Figure 3**).

Among these, there were 68 cases of invasive breast carcinoma, 12 cases of ductal carcinoma in situ, 3 cases of intraductal papillary carcinoma, and 1 case of mucinous carcinoma. The average size of the breast tumors was 2.38 ± 0.82 cm (**Table 1**). A total of 86 SLNs were identified using intradermal CEUS. Pathological examination confirmed 29 metastatic SLNs and 57 non-metastatic SLNs. On 2D US, 45 SLNs were identified as metastatic and 41 as non-metastatic. Intradermal CEUS identified 37 SLNs as metastatic and 49 as non-metastatic, whereas intravenous CEUS identified 33 SLNs as metastatic and 53 as non-metastatic. The average mean elasticity modulus (Emean), maximum elasticity modulus (Emax), and minimum elasticity modulus (Emin)values of the included 86 SLNs were 15.12 ± 5.36 kPa, 26.94 ± 19.07 kPa, and 7.54 ± 4.11 kPa, respectively. The average values for the transverse diameter, anteroposterior diameter, and cortical thickness were 1.41 ± 0.51 cm, 0.65 ± 0.22 cm, and 0.28 ± 0.14 cm, respectively.

**Table 1:** Basic characteristics of the included patients (n=84).

Variates	Patients
Age [year]	23–70
Mean age [mean ± s]	50.92 ± 10.08
BMI [kg/m^2^]	16.57–29.98
Mean BMI [kg/m^2^, mean ± s]	22.31 ± 3.14
Menopause status [number, %]	
Pre-menopause	32 (38.10%)
Post-menopause	52 (61.90%)
Tumor location [number, %]	
Outer upper quadrant	37 (44.05%)
Outer lower quadrant	18 (21.43%)
Inner lower quadrant	16 (19.05%)
Inner upper quadrant	10 (11.90%)
Central quadrant	3 (3.57%)
Tumor size [cm]	0.64-4.43
Mean tumor size [cm, mean ± s]	2.38 ± 0.82
Tumor type [number, %]	
Invasive breast carcinoma	69 (82.14%)
Ductal carcinoma in situ	15 (17.86%)

### 3.2 Comparison of the single-and multimodality ultrasounds in the diagnosis of metastatic SLNs

**Figure 4:**
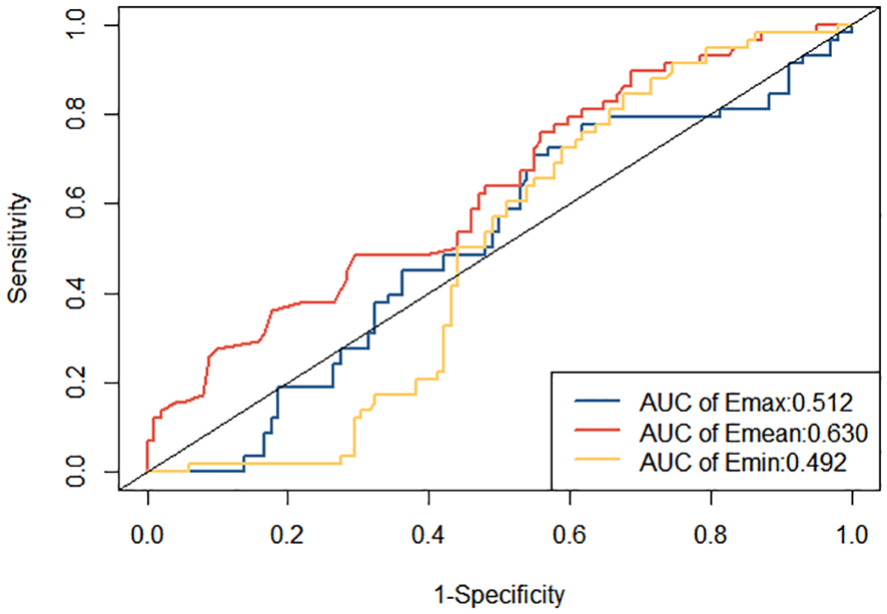
ROC curve for quantitative evaluation of metastatic SLNs in breast cancer using SWE.

**Figure 5:**
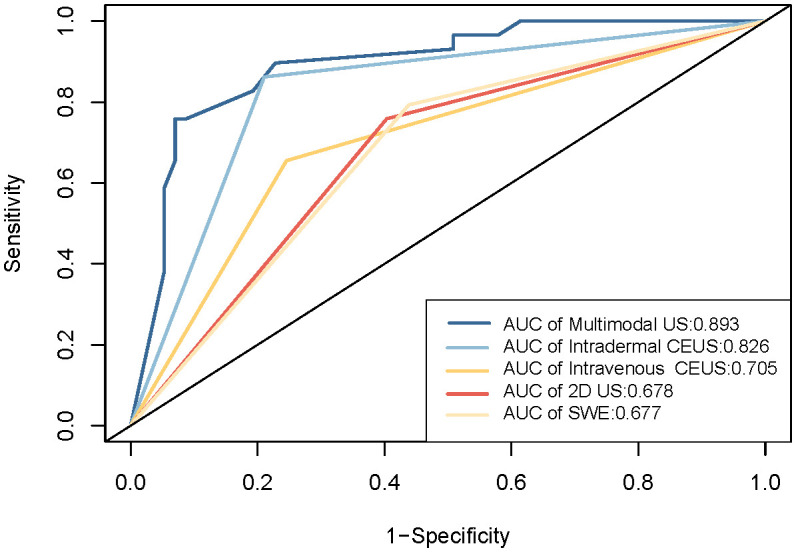
ROC curves for the diagnosis of metastatic SLNs using unimodal and multimodal ultrasound.

ROC curves were constructed to diagnose metastatic SLNs using the Emean, Emin, and Emax. The AUC values were 0.630 (95% confidence interval [95% CI] = 0.541–0.720) for Emean, 0.492 (95% CI = 0.403–0.501) for Emin, and 0.512 (95% CI = 0.420–0.605) for Emax (**Figure 4**). Therefore, using Emean > 14.3 kPa as the criterion for SWE, 38 cases were identified as metastatic SLNs and 48 cases were identified as non-metastatic SLNs. According to ROC curve analysis, in the single-modality diagnosis of SLN, the AUC values for intradermal CEUS, 2D US, intravenous CEUS, and SWE were 0.826 (95% CI = 0.743–0.909), 0.705 (95% CI = 0.600–0.809), 0.678 (95% CI = 0.576–0.780), and 0.677 (95% CI = 0.578–0.777), respectively (**Figure 5**, **Table 2**). The DeLong test revealed that the diagnostic performance of intradermal CEUS was significantly better than that of the other three single-modality ultrasound imaging techniques (P<0.05). Multimodal ultrasound consisting of subcutaneous CEUS, 2D US, intravenous CEUS, and SWE for diagnosing SLN achieved an AUC of 0.893 (95% CI = 0.821–0.964), with a sensitivity and specificity of 75.9% and 93.0%, respectively. In addition, the DeLong test demonstrated that the AUC of multimodal ultrasound was significantly higher than that of any of the single-modal ultrasound diagnostics (all P<0.05) as shown in **Table 2**.

**Table 2:** Efficiency of unimodal and multimodal ultrasound in diagnosing metastatic SLNs in breast cancer.

Method	Intradermal CEUS	Intravenous CEUS	2D US	SWE	Multimodal Ultrasound
AUC (95% CI)	0.826 (0.743–0.909)	0.705 (0.600–0.809)	0.678 (0.576–0.780)	0.677 (0.578–0.777)	0.893 (0.821-0.964)
Maximum Youden index	0.651	0.410	0.355	0.355	0.688
Sensitivity	0.862	0.655	0.759	0.793	0.759
Specificity	0.790	0.754	0.597	0.561	0.930
Positive predictive value	0.676	0.576	0.489	0.479	0.846
Negative predictive value	0.918	0.811	0.829	0.842	0.883
Positive likelihood ratio	4.095	2.667	1.880	1.808	10.810
Negative likelihood ratio	0.174	0.457	0.405	0.369	0.260

SLN, sentinel lymph node; CEUS, contrast-enhanced ultrasound; 2D US, two-dimensional ultrasound; SWE, shear-wave elastography; AUC, area under the curve.

**Figure 6:**
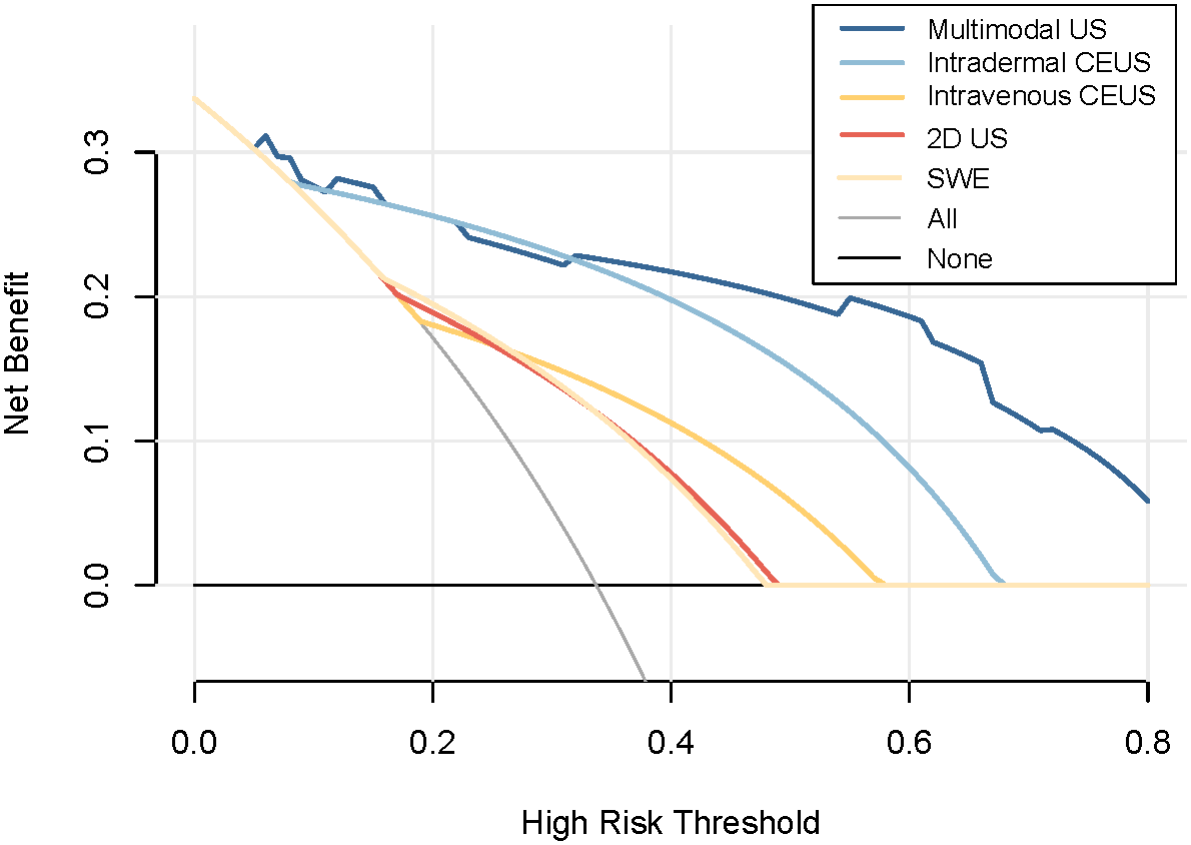
DCA curves for the diagnosis of metastatic SLNs using unimodal and multimodal ultrasound.

**Figure 7:**
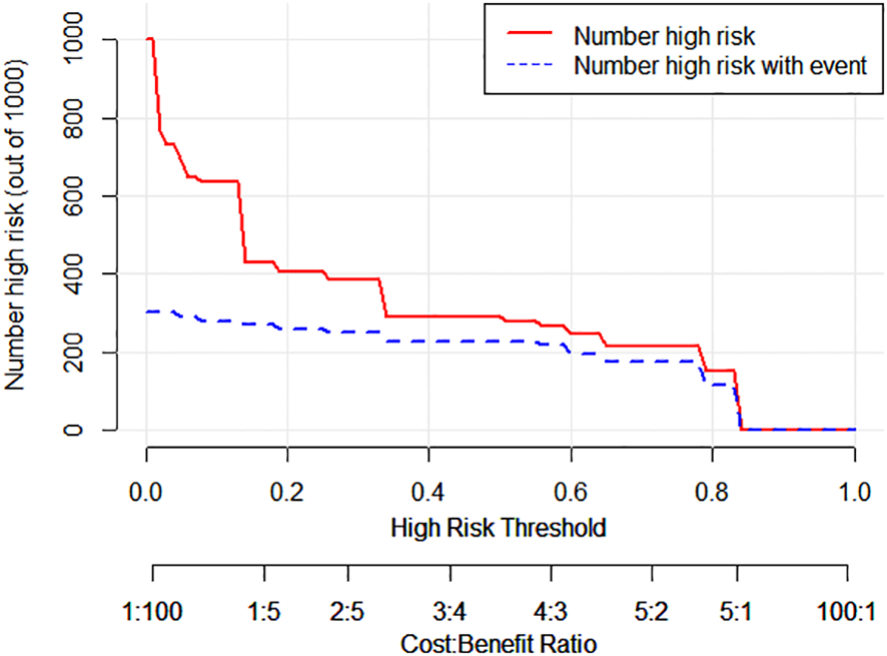
Cumulative impact curve for the diagnosis of metastatic SLNs using multimodal ultrasound.

The decision curve analysis (DCA) curve demonstrates that multimodal ultrasound outperforms the four single-modality ultrasound imaging techniques in terms of the probability threshold and net benefit. Furthermore, DCA indicated that multimodal ultrasound has higher clinical utility when the threshold probability is greater than 6%, suggesting its strong clinical practicality in such cases (**Figure 6**). The clinical impact curve demonstrated that multimodal ultrasound imaging can effectively identify high-risk breast cancer patients with SLNs, resulting in higher clinical utility (**Figure 7**).

## 4 Discussion

In patients with breast cancer, the SLN is the first lymph node to receive lymphatic drainage and it the most likely site of cancer cell metastasis. In recent years, 2D US, intradermal CEUS, intravenous CEUS, and SWE have become research hotspots for evaluating SLN characteristics in patients with breast cancer. Numerous studies have employed either single-modality ultrasound or a combination of two techniques for diagnosing metastatic SLNs (24–27). However, comparative studies evaluating the diagnostic performance of 2D US, intradermal CEUS, intravenous CEUS, and SWE as single-modality ultrasound techniques for metastatic SLNs and their assessment of SLN characteristics using multimodality ultrasound techniques are lacking.

In the comparative analysis of single-modality ultrasound in this study, intradermal CEUS demonstrated superior diagnostic efficacy for breast cancer SLNs compared to 2D US, intravenous CEUS, and SWE. Intradermal CEUS has a higher diagnostic value for the qualitative assessment of preoperative SLNs in breast cancer. This may be attributed to the following factors. In this study, most of the metastatic SLNs were in an early stage of the disease, with cancer cells primarily infiltrating the lymphatic vessels within the SLN capsule. At this stage, there may be no significant structural changes, and intradermal CEUS—which visualizes the lymphatic circulation—can detect tiny metastatic foci earlier and more sensitively, resulting in better diagnostic efficacy (28, 29). In contrast, 2D US may have limited diagnostic accuracy for early-stage metastatic SLNs when malignant features are not clear. When SLNs exhibit micrometastases, they may not induce the formation of new tumor blood vessels. As a result, some metastatic SLNs may still exhibit a branching pattern of enhancement radiating from the lymphatic hilum, making intravenous CEUS less sensitive for the early and accurate assessment of SLN properties (30). This could lead to a lower sensitivity. Additionally, factors such as inflammation, age, and depth from the body surface can influence the elasticity values obtained using SWE, limiting its utility (31, 32). Therefore, for patients with limited economic resources or poor compliance, intradermal CEUS alone may be a suitable choice for evaluating metastatic SLNs.

This study aimed to assess and enhance the diagnostic efficacy of SLN detection in breast cancer through a comprehensive comparison of single-modal ultrasound and various combinations of multimodal ultrasound approaches. The results of this study unequivocally demonstrated a significant improvement in diagnostic accuracy when all four ultrasound imaging modalities were synergistically employed in the multimodal ultrasound approach. Notably, this approach exhibited heightened specificity compared with the use of subcutaneous CEUS as a stand-alone modality. The primary driving factor behind this noticeable enhancement in diagnostic performance is the intricacies associated with subcutaneous CEUS. This ultrasound technique demands a higher level of operator expertise and precision as it entails precise control over factors such as injection depth and thorough massage at the injection site. Deviations from optimal procedures during subcutaneous injection of contrast agents can lead to incomplete drainage of the contrast agent into SLNs. This can result in false local perfusion defects or instances in which no contrast agent perfusion is observed. These limitations may compromise the specificity of subcutaneous CEUS when used in isolation to diagnose metastatic SLN. In contrast, the multimodal ultrasound approach addresses these challenges by capitalizing on the strengths of each imaging modality while offsetting their respective limitations. This holistic approach not only enhances diagnostic accuracy but also instills greater confidence in SLN diagnosis in patients with breast cancer. These findings underscore the potential clinical benefits of adopting a multimodal ultrasound strategy for more precise and reliable SLN detection, thereby advancing the management of breast cancer with improved diagnostic precision and patient care.

The analysis of DCA curves provides crucial insights into the outstanding performance of multimodal ultrasound in breast cancer SLN diagnosis. These curves clearly demonstrate that, in comparison with the other four single ultrasound imaging modalities, multimodal ultrasound performs more effectively across a range of probability thresholds, implying that it can offer a higher net clinical benefit in various clinical decision scenarios. Notably, when the probability threshold was set at approximately 6% or higher, multimodal ultrasound exhibited significant clinical utility, underscoring its robust practical potential for diagnosing high-risk breast cancer patients. Furthermore, the clinical impact curve emphasizes the diagnostic value of multimodal ultrasound. These curves clearly reveal the excellent performance of multimodal ultrasound imaging in discriminating high-risk breast cancer patients with SLNs, reflected in its high clinical yield. Multimodal ultrasound not only provides accurate diagnostic results, but also holds promise in offering physicians more informed decision support for more effective management and improved prognosis of breast cancer patients in clinical practice. These findings strongly support the prospects of multimodal ultrasound technology for breast cancer SLN diagnosis and underscore its significance in enhancing patient care and clinical decision-making. In addition, this study identified four cases that were pathologically confirmed as non-metastatic SLNs. These cases exhibited local perfusion defects on subcutaneous CEUS, which raised the suspicion of metastatic SLN. However, both the 2D US and SWE results indicated the presence of non-metastatic SLN. By considering the comprehensive findings of 2D US, subcutaneous CEUS, and SWE from the multimodal ultrasound approach, these cases were correctly classified as negative, effectively avoiding misdiagnosis and enhancing the qualitative diagnostic accuracy of the SLN. This finding underscores the importance of multimodal ultrasound in addressing complex scenarios for SLN diagnosis in patients with breast cancer. By combining different ultrasound imaging modalities, a more thorough assessment of the SLN status can be achieved, reducing the likelihood of misdiagnosis and providing clinicians with more reliable diagnostic information, thereby improving patient care and treatment decisions. This further emphasizes the potential application of multimodal ultrasound in breast cancer management, particularly in enhancing diagnostic efficacy when treating patients presenting with complex SLN.

Some limitations are acknowledged in the present study. The samples size was relatively small, further studies using larger sample sizes across multiple centers are needed. Moreover, the multimodality ultrasound and the CEUS are still not approved in the international guidelines for the study of breast cancer, and this method is not available in all breast cancer unit centers.

In conclusion, both single- and multimodal ultrasonography have clinical significance in the diagnosis of metastatic SLNs in breast cancer. Among single-modality ultrasound methods, intradermal CEUS showed the highest diagnostic value for metastatic SLNs in breast cancer. However, multimodal ultrasound demonstrated superior diagnostic efficacy for SLNs compared to single-modal ultrasound.

## Data availability statement

The raw data supporting the conclusions of this article will be made available by the authors, without undue reservation.

## Ethics statement

The studies involving humans were approved by the Ethics Committee of the Second Affiliated Hospital of Fujian Medical University. The studies were conducted in accordance with the local legislation and institutional requirements. The participants provided their written informed consent to participate in this study.

## Author contributions

SS: Conceptualization, Investigation, Writing – original draft. JY: Data curation, Formal Analysis, Software, Writing – review & editing. HK: Methodology, Project administration, Supervision, Writing – review & editing. HZ: Funding acquisition, Visualization, Writing – review & editing. GL: Writing – review & editing. ZX: Resources, Validation, Writing – review & editing.
